# Neuropsychopharmacology of Emerging Drugs of Abuse: *meta*- and *para*-Halogen-Ring-Substituted α-PVP (“*flakka*”) Derivatives

**DOI:** 10.3390/ijms23042226

**Published:** 2022-02-17

**Authors:** Núria Nadal-Gratacós, Esther Lleixà, Mónica Gibert-Serramià, Roger Estrada-Tejedor, Xavier Berzosa, Xavier Batllori, David Pubill, Jordi Camarasa, Elena Escubedo, Raúl López-Arnau

**Affiliations:** 1 Department of Pharmacology, Toxicology and Therapeutic Chemistry, Institute of Biomedicine (IBUB), Faculty of Pharmacy and Food Science, University of Barcelona, 08028 Barcelona, Spain; nuria.nadal@iqs.url.edu (N.N.-G.); estherlleixav@iqs.edu (E.L.); monicagibertserramia@gmail.com (M.G.-S.); d.pubill@ub.edu (D.P.); jcamarasa@ub.edu (J.C.); 2 Pharmaceutical Chemistry Group (GQF), IQS School of Engineering, Universitat Ramon Llull, 08017 Barcelona, Spain; roger.estrada@iqs.url.edu (R.E.-T.); xavier.berzosa@iqs.url.edu (X.B.); xavier.batllori@iqs.url.edu (X.B.)

**Keywords:** synthethic cathinones, new psychoactive substances, α-PVP, psychostimulant, reward, anxiety, structure-activity relationship

## Abstract

Changes in the molecular structure of synthetic cathinones has led to an increase in the number of novel emerging drugs in the illicit drug market at an unprecedented rate. Unfortunately, little is known about the neuropsychopharmacology of recently emerged halogen-substituted α-PVP derivatives. Thus, the aim of this study was to investigate the role of *para*- and *meta*-halogen (F-, Cl-, and Br-) substitutions on the in vitro, in silico, and in vivo effects of α-pyrrolidinopentiophenone (α-PVP) derivatives. HEK293 cells expressing the human dopamine or serotonin transporter (hDAT and hSERT) were used for the uptake inhibition and transporter affinity assays. Molecular docking was used to model the interaction mechanism against DAT. Swiss CD-1 mice were used for the horizontal locomotor activity, open field test, and conditioned place preference paradigm. All compounds demonstrated potent DA uptake inhibition and higher DAT selectivity than cocaine. *Meta*-substituted cathinones showed higher DAT/SERT ratios than their *para*- analogs, which correlates with an increased psychostimulant effect in vivo and with different *meta*- and *para*-in silico interactions at DAT. Moreover, all compounds induced rewarding and acute anxiogenic effects in mice. In conclusion, the present study demonstrates the role of *meta*- and *para*-halogen substitutions in the mechanism of action and provides the first evidence of the rewarding and anxiety-like properties of halogenated α-PVP derivatives.

## 1. Introduction

The global illicit drug market has changed remarkably over the last few years, and more than 400 new psychoactive substances (NPS) have been detected annually in Europe since 2015 [1], leading to an increase of the chemical diversity of NPS at an unprecedented rate. NPS are defined as substances of abuse, either in a pure form or a preparation, that are designed in order to mimic the effect of already considered illicit compounds. Moreover, new molecular entities are developed in order to replace the molecular structure of those NPS that have been banned. Among NPS, one of the most rapidly arising groups of compounds are synthetic cathinones [2]. The effects that they produce are characterized by stimulant, empathogenic, and euphoric properties, similar to those of methamphetamine and cocaine [3].

**Figure 1:**
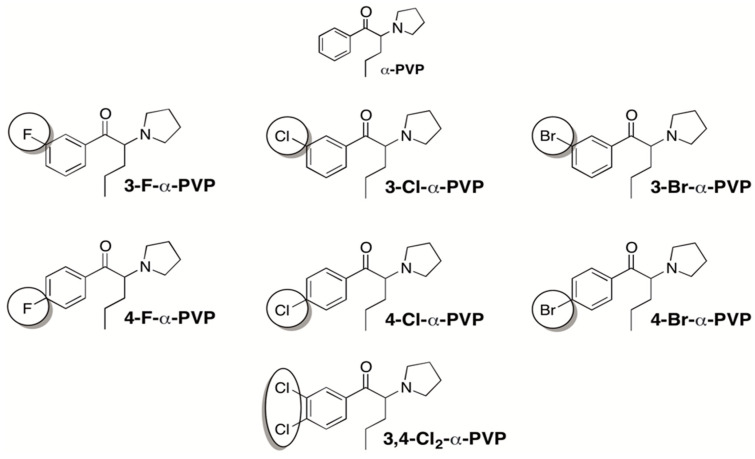
Chemical structure of α-PVP, 3-F-α-PVP, 4-F-α-PVP, 3-Cl-α-PVP, 4-Cl-α-PVP, 3,4-Cl_2_-α-PVP, 3-Br-α-PVP, and 4-Br-α-PVP.

Mephedrone (4-methyl-methcathinone) was one of the most commonly found cathinones in the illicit drug market in the early 2000s [4] and was shortly after banned in some countries and classified as Schedule I under the Controlled Substances Act in 2012 [5]. New alternatives derived from the chemical structure of mephedrone rapidly emerged, such as the halogen-derivatives Flephedrone (4-FMC), 3-Fluoromethcathinone (3-FMC), Clephedrone (4-CMC), 3-Chloromethcathinone (3-CMC), and Brephedrone (4-BMC) [6,7,8], which act as potent monoamine uptake inhibitors and releasers [9]. In fact, the use of novel halogenated synthetic cathinones recently became popular, likely due to their higher potency [10]. In a similar manner, α-Pyrrolidinopentiophenone (α-PVP), more commonly known as “*flakka*”, is one of the most popular synthetic cathinones in the United States and Europe [11,12]. The structure of α-PVP is similar to that of 3,4-methylenedioxypyrovalerone (MDPV), which only differs in the presence of the 3,4-methylenedioxy substitution. Both MDPV and α-PVP have been reported to act as potent dopamine (DA) but weak serotonin (5-HT) reuptake inhibitors [13,14,15,16]. Previous studies have also reported the important long-lasting psychomotor stimulant, rewarding, and reinforcing properties of α-PVP [14,17,18]. The increased popularity of this cathinone led to overdose cases, intoxications, and even fatalities related to its consumption [19,20,21]. In 2014, α-PVP was temporarily classified by the Drug Enforcement Administration (DEA) as a Schedule I compound [22], and since then new derivatives have emerged to replace it. Therefore, with the endeavor to identify and evaluate potential alternatives to α-PVP, as well as to study the structure-activity relationship (SAR) of halogen-substituted analogs of α-PVP, this study is focused on seven novel synthetic cathinones: 3-Fluoro-α-Pyrrolidinopentiophenone (3-F-α-PVP), 4-Fluoro-α-Pyrrolidinopentiophenone (4-F-α-PVP), 3-Chloro-α-Pyrrolidinopentiophenone (3-Cl-α-PVP), 4-Chloro-α-Pyrrolidinopentiophenone (4-Cl-α-PVP), 3,4-Dichloro-α-Pyrrolidinopentiophenone (3,4-Cl_2_-α-PVP), 3-Bromo-α-Pyrrolidinopentiophenone (3-Br-α-PVP), and 4-Bromo-α-Pyrrolidinopentiophenone (4-Br-α-PVP) (Figure 1).

The use and abuse of *para*-halogenated α-PVP derivatives has already been reported [23,24,25,26,27,28,29]. In fact, 4-Cl-α-PVP was temporary classified as a Schedule I compound in 2019 [30]. Studies reported by [31,32] have demonstrated that *para*-halogenated α-pyrrolidinophenones act as potent DA uptake inhibitors. Regarding the in vivo effects, the authors of [33] recently demonstrated that 4-F-α-PVP administration induces a dose-dependent increase in horizontal locomotor activity in mice. Additionally, 3-F-α-PVP and 3-Br-α-PVP are also available through the internet, and some samples have been identified in non-governmental organizations’ (NGOs’) drug testing laboratories (i.e., www.drugsdata.org; www.ecstasydata.org, accessed on 20 December 2021); although, to our knowledge, there is no information regarding the pharmacology of these *meta*-halogenated derivatives. Finally, 3-Cl-α-PVP and 3,4-Cl_2_-α-PVP were also selected in this study due to the similarity that they present with the already available halogen-derivatives of other novel synthetic cathinones and for the high affinity at DAT that has been previously reported for 3,4-Cl_2_-α-PVP [31]. However, little is known regarding the in vitro, in silico, and in vivo neuropharmacology of these novel synthetic cathinones.

Thus, the aim of this study was to (i) synthesize and characterize seven α-PVP derivatives; (ii) study their mechanism of action by means of uptake inhibition, transporter binding assays, and molecular docking; (iii) assess in vivo their psychostimulant, anxiogenic, and rewarding properties. This study also intends to provide an SAR between halogen ring-substitutions of synthetic cathinones, which may offer valuable information to predict the neuropharmacological effects of similar cathinones that may appear in the near future.

## 2. Results

### 2.1. Effect of α-PVP Derivatives on Monoamine Uptake Inhibition in Transfected HEK293 Cells

**Figure 2:**
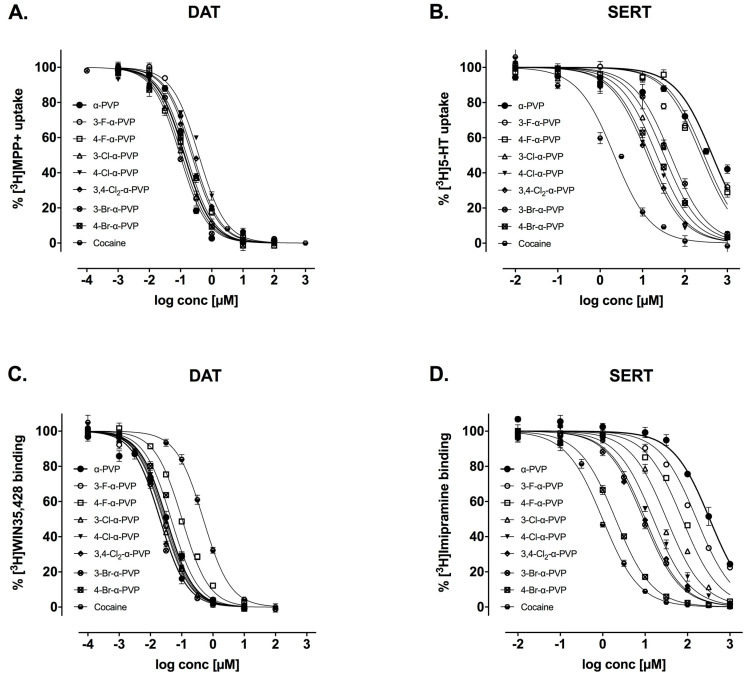
Concentration-effect curves of α-PVP, 3-F-α-PVP, 4-F-α-PVP, 3-Cl-α-PVP, 4-Cl-α-PVP, 3,4-Cl_2_-α-PVP, 3-Br-α-PVP, 4-Br-α-PVP, and cocaine on [^3^H]MPP+ uptake and [^3^H]WIN35,428 binding at DAT (Panel (**A**,**C**)) and [^3^H]5-HT uptake and [^3^H]Imipramine binding at SERT (Panel (**B**,**D**)) in transfected HEK293 cells. Data are expressed as a percentage of control uptake (mean ± SEM) of six independent experiments performed per triplicate.

All cathinones showed potent DA uptake inhibition, presenting low IC_50_ values for hDAT, while exhibiting a much lower activity at hSERT. The corresponding IC_50_ values and hDAT/hSERT inhibition ratios are summarized in Table 1, and concentration-response curves are depicted in Figure 2A,B. 3-F-α-PVP, 4-F-α-PVP, 3-Br-α-PVP, and 4-Br-α-PVP showed a similar capacity to inhibit [^3^H]MPP^+^ uptake at hDAT to α-PVP and are 2-fold more potent at inhibiting DAT than cocaine. While still presenting an important monoamine uptake inhibition at DAT, 3-Cl-α-PVP and 4-Cl-α-PVP are, among the cathinones tested, the ones with higher IC_50_ values for DAT. Regarding hSERT inhibition, the cathinones substituted with F-, along with α-PVP, were the ones with a lower inhibition potency.

### 2.2. Effect of α-PVP Derivatives on Transporter Binding Affinities

The binding affinity constants of the tested drugs (*Ki*) for DAT and SERT are summarized in Table 1. All the synthetic cathinones tested have shown greater affinity to DAT than cocaine. Specifically, the synthetic cathinones substituted in *para*- seem to have less affinity compared to the ones substituted in *meta*- position.

Even though all drugs presented a substantially low affinity to SERT, 3-F-α-PVP, 4-F-α-PVP, and α-PVP were the ones with the lowest affinity. Finally, the hDAT/hSERT affinity ratio is calculated and summarized in Table 1, and concentration-response curves are presented in Figure 2C,D.

### 2.3. Molecular Docking of α-PVP Derivatives at hDAT

**Figure 3:**
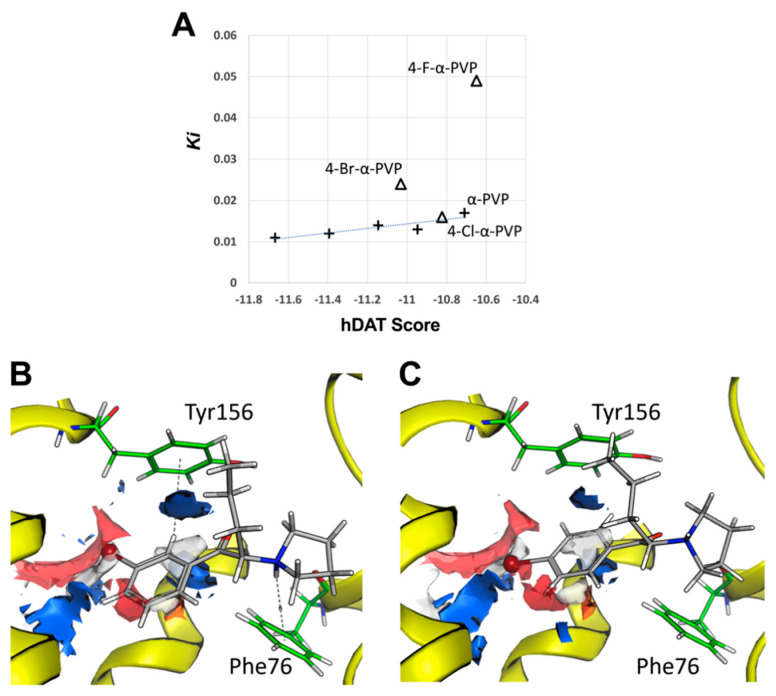
(**A**) Comparison between experimental Ki values and the affinity predicted by molecular docking for all studied compounds. Determination coefficient R^2^ = 0.805 and Spearman’s rank correlation coefficient ρ = 0.81. (**B**,**C**) An illustrative example (3-Br-α-PVP and 4-Br-α-PVP) of the interaction mechanism predicted. Electrostatic map is shown describing the preferred interacting regions with H-bond acceptors (red), H-bond donors (blue), and hydrophobic regions (white).

The binding mechanism for the cathinones under study has been assessed by means of molecular docking. Binding affinities predicted by docking show a remarkable correlation with Ki experimental values for *meta*-substituted derivatives (R^2^ = 0.8) (Figure 3A). However, *para*-substituted derivatives break the trend, suggesting a modification on the binding mechanism resulting from the change in halogen position, which would not be properly described by the scoring function. In general, the description of halogen bonding is not consistently described in scoring functions requiring in-depth study [34]. *Meta-*substituted compounds show a similar binding mechanism, H-π interactions being the most prevalent. An H-π interaction is stablished by the ring supporting the halogen with Tyr156 and another interaction is stablished between the heterocyclic ring and Phe76 (Figure 3B). Electrostatic maps reveal that the halogen atom in *meta*-substituted cathinones is faced towards the Tyr156 region, located in an electrophilic region, facilitating the stabilization of the complex through halogen bonding (e.g., bromine atom in 3-Br-α-PVP is located at 2.7 Å from Ala423, Figure 3B). However, results obtained for the *para*-substituted compounds show the loss of Tyr156 interaction and the relocation of the halogen to a neutral region of the active site, in terms of electrostatics, where no halogen bond can be stablished (Figure 3C).

### 2.4. Effect of α-PVP Derivatives on HLA

**Figure 4:**
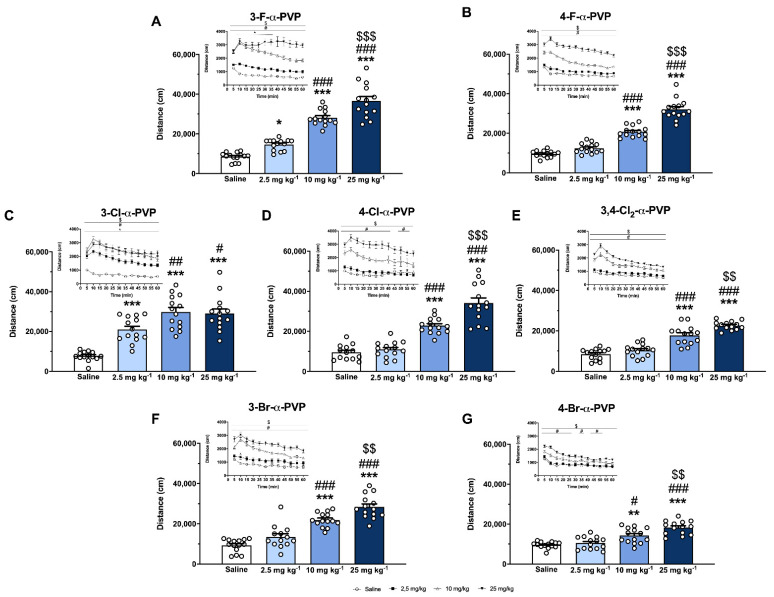
Effects of halogenated α-PVP derivatives on cumulative locomotor activity in CD-1 mice and its time course profile (inlet). For HLA, bars represent mean ± SEM of the distance travelled in 60 min. * *p* < 0.05, ** *p* < 0.01, and *** *p* < 0.001 vs. saline, # *p* < 0.05, ## *p* < 0.01, and ### *p* < 0.001 vs. 2.5 mg kg^−1^, $$ *p* < 0.01 and $$$ *p* <0.001 vs. 10 mg kg^−1^. For HLA time course profile, each time point represents mean ± SEM of the distance (in cm) travelled in 5 min blocks. Only comparisons vs. the corresponding saline group and *p* < 0.5 are shown for clarity purposes. * *p* < 0.05 2.5 mg kg^−1^ dose group vs. saline, # < 0.05 10 mg kg^−1^ dose group vs. saline, $ *p* < 0.05 30 mg kg^−1^ dose group vs. saline. Panel (**A**) saline and 3-F-α-PVP, N = 14/group. Panel (**B**) saline and 4-F-α-PVP, N = 14/group. Panel (**C**) saline and 3-Cl-α-PVP, N = 14/group. Panel (**D**) saline, N = 13/group and 4-Cl-α-PVP, N = 14/group. Panel (**E**) saline and 3,4-Cl_2_-α-PVP, N = 14 /group. Panel (**F**) saline, 3-Br-α-PVP 10 mg kg^−1^ and 3-Br-α-PVP 25 mg kg^−1^, N = 14/group; 3-Br-α-PVP 2.5 mg kg^−1^, N = 13/group. Panel (**G**) saline and 4-Br-α-PVP, N = 14/group.

As shown in Figure 4, all compounds increased HLA in a dose-dependent manner in mice. For statistical results, see Supplemental Material Table S1. A significant increase in HLA for all substances after 10 and 25 mg kg^−1^ injections compared to the saline group was observed. Moreover, 3-Cl-α-PVP was the only cathinone tested that revealed a ceiling effect after 25 mg kg^−1^ injection. 3-F-α-PVP and 3-Cl-α-PVP were the only drugs to show a significant effect after 2.5 mg kg^−1^ compared to the saline group.

In order to analyze any difference in a compound’s efficacy, comparisons between compounds at the same dose tested was performed (Figure S1). Specifically, when analyzing the HLA induced by all the compounds at the medium dose tested (10 mg kg^−1^) (Figure S1B), a significant higher efficacy, at such dose, for the *meta*-substituted cathinones than their *para*-substituted analogs was observed.

HLA time courses are also shown in Figure 4 (inlet). The statistical results of the two-way ANOVA of repeated measures applied are presented in the Supplemental Material Table S1. HLA profiles revealed a rapid onset effect (5–10 min) for all the compounds after 10 and 25 mg kg^−1^ injections. Moreover, at the medium dose tested, the increase in locomotor activity ended before a 60 min period for 4-Cl-α-PVP and 4-Br-α-PVP. However, at 25 mg kg^−1^, the psychostimulant effect lasted for more than 60 min for all the cathinones tested.

### 2.5. Effect of α-PVP Derivatives on Anxiety-like Behavior

**Figure 5:**
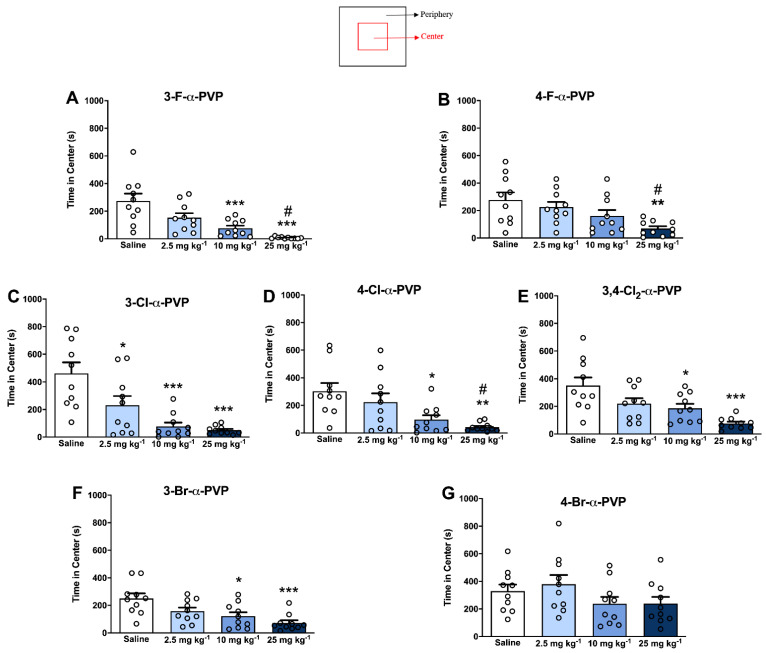
Effects of 3-F-α-PVP (**A**), 4-F-α-PVP (**B**), 3-Cl-α-PVP (**C**), 4-Cl-α-PVP (**D**), 3,4-Cl_2_-α-PVP (**E**), 3-Br-α-PVP (**F**), and 4-Br-α-PVP (**G**) (2.5, 10 and 25 mg kg^−1^, i.p.) on Open Field test (anxiety-like behavior) in CD-1 mice. Bars represent mean ± SEM of time in center. N = 10/group. * *p* < 0.05 vs. saline and # <0.05 vs. 2.5 mg kg^−1^. * *p* < 0.05, ** *p* < 0.01, and *** *p* < 0.001 vs. saline, # *p* < 0.05 vs. 2.5 mg kg^−1^.

The time spent in the center of the open-field arena is depicted in Figure 5, and statistical results are presented in Table S2. Except for 4-Br-α-PVP, a significant dose-dependent decrease in the time that animals spent in the center of the arena was observed for all the rest of the cathinones tested, suggesting that they can induce acute anxiogenic effects. Particularly, the highest anxiogenic potency was found in mice administered with 3-Cl-α-PVP, which were the only ones showing anxiogenic effects at the lowest dose tested (2.5 mg kg^−1^); whereas the mice treated with 4-F-α-PVP only showed a decrease in the time spent in the center at the highest dose tested.

### 2.6. Effect of α-PVP Derivatives on CPP

**Figure 6:**
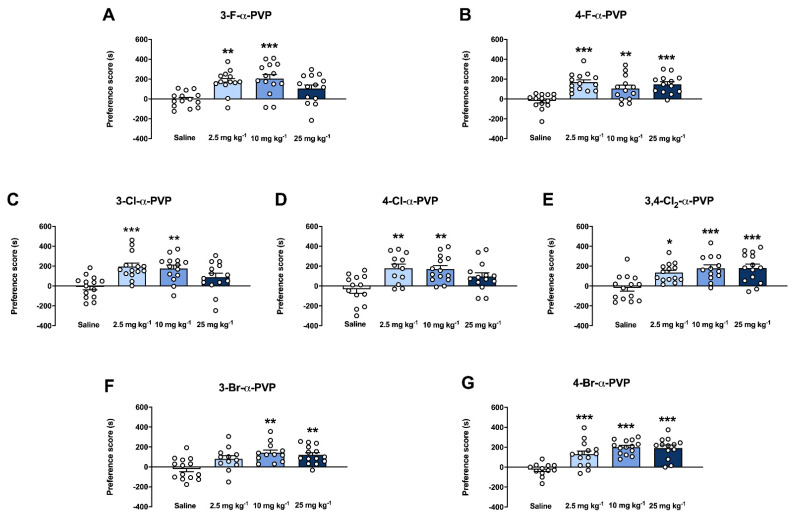
Effects of halogenated α-PVP derivatives on CPP test in CD-1 mice. Bars represent mean ± SEM of the preference score (difference between the time spent in the drug-paired compartment on the test day and the pre-conditioning day). Panel (**A**) saline, 3-F-α-PVP 10 mg kg^−1^, and 3-F-α-PVP 25 mg kg^−1^, N = 14/group and 3-F-α-PVP 2.5 mg kg^−1^, N = 13/group. Panel (**B**) saline and 4-F-α-PVP 2.5 mg kg^−1^, N = 14/group; 4-F-α-PVP 10 mg kg^−1^ and 4-F-α-PVP 25 mg kg^−1^, N = 13/group. Panel (**C**) saline and 3-Cl-α-PVP, N = 14. Panel (**D**) saline, N = 13/group; 4-Cl-α-PVP 2.5 mg kg^−1^, N = 12/group, 4-Cl-α-PVP 10 mg kg^−1^, and 4-Cl-α-PVP 25 mg kg^−1^, N = 14/group. Panel (**E**) saline, 3,4-Cl_2_-α-PVP 2.5 mg kg^−1^, and 3,4-Cl_2_-α-PVP 25 mg kg^−1^, N = 14/group and 3,4-Cl_2_-α-PVP 10 mg kg^−1^, N = 13/group. Panel (**F**) saline and 3-Br-α-PVP 25 mg kg^−1^, N = 14, 3-Br-α-PVP 2.5 mg kg^−1^ and 3-Br-α-PVP 10 mg kg^−1^, N = 12/group. Panel (**G**) saline, N = 12/group, 4-Br-α-PVP 2.5 mg kg^−1^ and 4-Br-α-PVP 10 mg kg^−1^, N = 14/group and 4-Br-α-PVP 25 mg kg^−1^, N = 13/group. * *p* < 0.05, ** *p* < 0.01 and *** *p* < 0.001 vs. saline.

The rewarding effects of 3-F-α-PVP, 4-F-α-PVP, 3-Cl-α-PVP, 4-Cl-α-PVP, 3,4-Cl_2_-α-PVP, 3-Br-α-PVP, and 4-Br-α-PVP were studied using the CPP paradigm. Nine animals were withdrawn from the experiments due to an initial preference for one of the compartments (>70% of the total session time). For statistical results, see Supplemental Material Table S3. As depicted in Figure 6, all compounds induced a significant increase in the preference score at 10 mg kg^−1^ compared to their corresponding saline-treated group. 3-Br-α-PVP was the only cathinone that did not show rewarding properties at the lowest dose tested (2.5 mg kg^−1^). Moreover, 4-F-α-PVP, 3,4-Cl_2_-α-PVP, 3-Br-α-PVP, and 4-Br-α-PVP also showed a significant increase of the rewarding effects at the highest dose tested (25 mg kg^−1^).

## 3. Discussion

Given the powerful psychostimulant and addictive effects that synthetic cathinones produce in humans, along with the evidence that the illicit drug market continues to synthetize and distribute novel synthetic cathinone analogs, it is becoming imperative to study how different structural modifications can influence their in vitro and in vivo effects, which can aid in predicting the abuse potential, pharmacology, and toxicology of novel synthetic cathinones.

Because pyrrolidine-containing cathinones are described as acting as monoamine transporter inhibitors [31,35], we first focused on the ability of α-PVP derivatives to act as DA and 5-HT uptake inhibitors. Our results demonstrated that all halogenated pyrrolidinophenones tested act as potent and selective DA uptake inhibitors. Previous studies have also reported a direct relationship between high DAT/SERT ratios and abuse liability [36]. In our study, all α-PVP derivatives tested showed higher DAT/SERT inhibition and affinity ratios than cocaine, which may suggest a high abuse potential.

Regarding SAR, in both uptake and binding experiments, *para*- substitutions slightly decreased the potency inhibiting DA uptake as well as the DAT affinity. Moreover, the halogenated *meta*-substituted cathinones tested appear to be more DAT selective than their *para*-analogs. This SAR seems to be a common feature among synthetic cathinones because other authors also reported that 3-substituted analogs of methcathinone are slightly more DAT selective than the corresponding 4-substituted compounds [37]. Although a direct correlation between the volume of the halogen and the ability to inhibit DA uptake or the DAT/SERT inhibition ratio was not observed, our data showed an increased DAT/SERT affinity ratio when decreasing the volume of the ring-substitution in *para*- and *meta*-substituted pyrrolidinophenones, as compounds with greater steric bulk did exhibit lower selectivity for DAT. This is in accordance with results at SERT reported by [38]. In fact, the addition of a halogen increased the potential to inhibit 5-HT uptake and the affinity towards SERT. This correlates with previous findings in which halogen-substituted amphetamine and methcathinone derivatives resulted in a stronger serotonergic potency [39,40]. Moreover, data reported by [31,32] also demonstrated that DAT selectivity decreases when increasing the volume of the halogen of α-PVP derivatives from F- to Cl- or Br- substitutions, respectively. Other studies have also demonstrated that the volume of *para*-substituents played an important role in the potency and selectivity of cathinones towards DAT and SERT [36]. Finally, despite no remarkable difference in the DA uptake inhibition potency and DAT affinity between mono- (3-Cl- and 4-Cl-) and di-substituted (3,4-Cl-) pyrrolidinophenone derivatives, 3,4-Cl-α-PVP possess a lower selectivity towards DAT than its *meta*-analog, but similar to its *para*-analog.

Experimental *Ki* values have been considered as reference when discussing the binding affinities predicted by molecular docking in in silico studies. In fact, our research group has recently demonstrated that the pyrrolidine group of α-PVP renounced to interact with Asp79 (DAT) when comparing to other amino-substituted cathinones, supporting the experimental data obtained concerning its potency and mechanism of action [14]. In the present study, computational results revealed that *para*-substituted compounds lose the interaction with Tyr156 (DAT) and the relocation of the halogen to a neutral region of the active site, in terms of electrostatics, where no halogen bond can be stablished. This differential binding mechanism of *meta*- and *para*-substituted cathinones may shed light on the different affinity and potency observed in vitro.

The psychostimulant properties of the seven cathinones have been assessed in a motor performance test, showing a time- and dose-dependent stimulation on locomotor activity for all of them. Specifically, the HLA results obtained for 4-F-α-PVP are in accordance with those recently reported by [33] using a similar dose range (5, 10, and 20 mg kg^−1^) in mice. However, little is known about the in vivo effects of most of the halogenated cathinones tested in this study. Moreover, 3-F- and 3-Cl-α-PVP induced hyperlocomotion at the lowest dose tested (2.5 mg kg^−1^), while the other compounds did not. In fact, when studying the efficacy at the medium dose tested (10 mg kg^−1^), our results revealed higher psychostimulant effects of *meta*-ring-substituted than their *para*-ring-substituted analogs. This in vivo finding also correlates with the in vitro data previously mentioned, in which a slight increase in DA uptake inhibition and DAT selectivity in *meta*-analogs was observed. However, this does not apply to all the compounds when analyzing the efficacy at the other doses tested, probably due to the fact that 2.5 mg kg^−1^ is a low dose not able to induce hyperlocomotion in most of the compounds, and 25 mg kg^−1^ is a very high dose that may produce other behavior altering locomotion (i.e., stereotypies, distress, etc.), as well as induce longer-lasting effects (>60 min).

Rodents typically spend a significantly greater amount of time exploring the periphery of the arena than the unprotected center area as they display a natural aversion to brightly lit open areas. This behavior is linked to anxiety related behaviors [41], yet, rodents also have a drive to explore a perceived threatening stimulus. Indeed, decreased levels of anxiety lead to increased exploratory behavior. Therefore, increased anxiety will result in a preference to stay in the outer edge of the arena. Thus, in our work, the open field test has been used to study the anxiety-like effects of halogen-derivatives of α-PVP as it has already been described for some synthetic cathinones [42,43]. Our results point to an anxiogenic effect induced by the acute administration of 3-F-α-PVP, 4-F-α-PVP, 3-Br-α-PVP, 3-Cl-α-PVP, 4-Cl-α-PVP, and 3,4-Cl_2_-α-PVP. Although the exact mechanism by which some psychostimulants induce acute anxiogenic-like behavior is not completely elucidated, there are growing evidences suggesting that dopaminergic mechanisms are involved in some aspects of anxiety [44,45,46]. In fact, acute administration of classical drugs of abuse that produce potent DA uptake inhibition, such as amphetamine, methamphetamine, and cocaine, has been demonstrated to be able to induce acute anxiogenic effects in rodents [47,48,49]. Moreover, changes in other neurotransmitters systems in the brain, such as norepinephrine and serotonin, may also be involved in anxiety behaviors [46]. It must be pointed out that 4-Br-α-PVP, the cathinone derivative with a lower hDAT/hSERT ratio, was the only one tested that did not cause acute anxiogenic effects, whilst displaying lower psychostimulant properties. In fact, it seems that *para*-analogs, which showed lower DAT selectivity versus SERT in comparison with *meta*-analogs, need higher doses to induce significant anxiogenic effects. Because selective 5-HT uptake inhibitors (SSRIs) have been demonstrated to be useful in anxiety disorders, such decrease in DAT selectivity versus SERT may explain the reduced efficacy at inducing anxiogenic effects.

Since the appearance of synthetic cathinones in the illicit drug market, several studies have demonstrated the rewarding and reinforcing properties of this NPS’s subclass in rodents; for a review, see [50]. However, to our knowledge, there is no information regarding the rewarding properties of the halogen-derivatives of α-PVP tested in this study. On one hand, our results demonstrated for the first time that all halogenated-α-PVP derivatives tested showed rewarding effects at least at two of the doses tested. This is in accordance with the high DAT/SERT inhibition and affinity ratios observed in vitro (from 38 to 1819 and from 52 to 4540, respectively). However, it must be pointed out that since in place conditioning, an all-or-nothing effect is observed [51,52], it is difficult to perfectly correlate such behavioral response with a quantitative in vitro result. On the other hand, all the compounds produced an increase in the preference score in the CPP paradigm at the medium dose tested (10 mg kg^−1^), revealing similar rewarding effects to cocaine at the same dose tested and methodological conditions, as described previously by our research group [53]. Moreover, while 3- and 4-Cl-α-PVP did not induce significant rewarding effects at the highest dose tested (25 mg kg^−1^), repeated exposure to 3,4-Cl-α-PVP at the same dose did, pointing to a particular and different contribution of the di-substitution in the rewarding effects induced by halogenated-pyrrolidinophenone derivatives.

Finally, while the DA uptake inhibition IC_50_ value and the affinity constant of cocaine for DAT are very similar, all α-PVP derivatives showed greater affinity constants for DAT (3–30-fold, approximately) than DA uptake inhibition potency, especially in chloro-ring derivatives. This fact may be in accordance with the proposed hypothesis of two discrete inhibitor-binding DAT conformations/populations in which the DAT conformation/population responsible for inhibitor high-affinity binding is less responsible for DA uptake [54,55]. According to that, the synthetic cathinones tested in this study displace [^3^H]WIN35428 at a DAT conformation/population unrelated to that at which they primarily inhibit DA uptake. This is of great importance because the development of potential “anti-cocaine” medications has been focused on compounds capable of binding to DAT with high affinity, hindering cocaine-binding, while not displaying abuse potential [56,57]. Moreover, we cannot rule out the role/implication of the recently reported mechanism of action of α-PVP as a pseudo-irreversible non-competitive inhibition in the divergence observed between DAT affinity and DA uptake inhibition [58] as well as its relation in the development of novel “anti-cocaine” compounds. Therefore, the in vitro results obtained in the present study may help and be used as a starting point for the study and design of novel and potent DAT inhibitors but with clinical utility (i.e., without abuse potential), which warrants further investigation.

## 4. Materials and Methods

### 4.1. Subjects

Male Swiss CD-1 mice (Janvier, Le Genest, France) weighing 30–35 g (6–8 weeks old) were randomly assigned to an experimental group. The animals were housed (seven per cage) in polycarbonate cages with wood-derived bedding, in temperature-controlled conditions (22 ± 1 °C) under a 12 h light/dark cycle and had ad libitum access to food (standard laboratory diet, Panlab SL, Barcelona, Spain) and drinking water. All animal care and experimental protocols in this study were approved by the Animal Ethics Committee of the University of Barcelona under the supervision of the Autonomous Government of Catalonia and are in accordance with the guidelines of the European Community Council (2010/63/EU), as amended by Regulation (EU) 2019/1010. Efforts were made to minimize animal use and suffering. All studies involving animals comply with the ARRIVE guidelines [59]. The animals were supervised before and throughout the study, immediately after injection, and during the behavioral experiment as well as 24 h after injection, and different parameters were visually evaluated for humane endpoints, such as abnormal posture, greater loss than 20%, self-mutilations, and strange vocalizations.

### 4.2. Drugs and Materials

Ring-substituted α-PVP derivatives were synthetized in racemic form as hydrochloride salts and characterized as described in the Supplemental Material. Solutions for injection were prepared daily in isotonic saline solution (0.9% NaCl, pH 7.4). [^3^H]1-Methyl-4-phenylpyridinium ([^3^H]MPP^+^), [^3^H]5-HT, [^3^H]WIN35,428, and [^3^H]imipramine were purchased from Perkin Elmer Inc. (Boston, MA, USA). HEPES sodium (HEPES-Na) and cell culture media (Dulbecco’s Modified Eagle’s medium (DMEM) high-glucose) were purchased from Sigma-Aldrich. Cocaine was provided by the Spanish National Institute of Toxicology. All other reagents were of analytical grade and purchased from several commercial sources.

### 4.3. Uptake Inhibition and Transporter Binding Assays in HEK293 Cells

#### 4.3.1. Cell Culture and Membrane Preparation

For the binding experiments and uptake inhibition assays, human embryonic kidney (HEK293) cells (CLS Cat# 300192/p777_HEK293, RRID:CVCL_0045) stably expressing the human isoforms of the transporters DAT and SERT were used. The generation and maintenance of stable, monoclonal cell lines expressing DAT and SERT was conducted as described by [60]. HEK293 were maintained in DMEM supplemented with heat-inactivated 10% FBS, 1 µg mL^−1^ streptomycin, and 100 U mL^−1^ penicillin and cultured to a subconfluential state in a humidified atmosphere (5% CO_2_, 37 °C). In order to maintain the selection process, Geneticin (G418; 50 µg mL^−1^) was added.

For the uptake inhibition assays, HEK293 cells expressing DAT or SERT were seeded onto 96-well plates, at a density of 0.36 million cells per well, previously treated (24 h) with poly-D-lysine (PDL).

To prepare membranes, HEK293 cells expressing monoamine transporters were harvested from 80–90% confluent dishes. In short, cells were washed with ice-cold phosphate buffered saline (PBS) twice. An extra 5 mL of PBS was added, and cells were mechanically detached using a plastic scraper and subsequently pelleted by centrifugation (400× *g* for 10 min at 4 °C). The resulting pellet was resuspended in hypotonic HME buffer (20 mM HEPES NaOH, 1 mM EDTA, 2 mM MgCl_2_; pH 7.4) and then subjected to two freeze-thaw cycles in liquid nitrogen and homogenization through sonication at 4 °C. Membranes were collected by centrifugation (40,000× *g* for 30 min at 4 °C) and resuspended in an appropriate volume of HME buffer. Protein concentration was determined using the Bio-Rad Protein Reagent (Bio Rad Laboratories, Hercules, CA, USA). Membrane preparations were kept at −80 °C.

For both uptake inhibition assays and transporter binding assays, α-PVP and cocaine were used as reference drugs.

#### 4.3.2. Uptake Inhibition Assays

The uptake inhibition assay was performed as described by [14,61], with minor modifications. Prior to starting the uptake inhibition experiment, the media was aspirated from the cell culture in the 96-well plates and immediately replaced with 200 µL of Krebs-HEPES-Buffer (KHB; 10 mM HEPES, 120 mM NaCl, 3 mM KCl, 2 mM CaCl_2_·2H_2_O, 2 mM MgCl_2_·6H_2_O supplemented with 20 mM D-glucose; pH 7.3). A preincubation phase, in which the cells are incubated for 5 min (hDAT, hSERT) with different concentrations of the cathinone in KHB at a final volume of 50 µL well^−1^, is required to ensure equilibration conditions. Subsequent to removing the preincubation solution, cells were incubated with the radiolabeled compounds, 0.02 µM [^3^H]MPP^+^ for hDAT and 0.1 µM [^3^H]5-HT for hSERT, together with different concentrations of the drug in KHB. The uptake incubation times were 3 min for hDAT and 1 min for hSERT. As the incubation time finished, the incubation solution was aspirated, and the cells were washed with ice-cold KHB and lysed with sodium dodecyl sulfate (SDS) 1%. The lysate was added to scintillation fluid and the radioactivity was quantified with a beta-scintillation counter (Perkin Elmer, Waltham, MA, USA). Parallel samples of non-specific uptake were determined in the presence of 100 µM of cocaine for hDAT and 30 µM of paroxetine for hSERT. The non-specific uptake value was <10% of the total uptake and was subtracted from the data to yield specific uptake, as described above. The uptake in the absence of the tested drugs was normalized to 100%, and the uptake in the presence of different concentrations of drugs was expressed as a percentage thereof. All determinations were performed per triplicate to ensure the reliability of single values (N = 6).

#### 4.3.3. Transporter Binding Assays

The drugs under study were dissolved in binding buffer (120 mM NaCl, 3 mM KCl, 2 mM MgCl_2_, 10 μM ZnCl_2_, and 20 mM Tris pH 7.4 for hDAT, and 120 mM NaCl, 3 mM KCl, 2 mM MgCl_2_, 1 mM EDTA, and 20 mM Tris pH 7.4 for hSERT) at a range of concentrations from 0.1 nM–1 M. Membrane preparations expressing the monoamine transporters hDAT and hSERT were incubated with tritiated selective ligands at concentrations close or equal to K_d_, and ligand displacement by the tested drugs at different concentrations was measured per triplicate to ensure reliability of single values (N = 6). The binding assays are performed in tubes containing 25 µL of the radiolabeled ligand ([^3^H]WIN35,428, K_d_ = 12 nM; B_max_ = 6.75 pmol mg^−1^ [62] (hDAT assay, final concentration 10 nM) or [^3^H]imipramine, K_d_ = 4.5 nM; Bmax = 15 pmol mg^1^ [59] (hSERT assay, final concentration 3 nM)) diluted in the corresponding reaction buffer, 15 µg of membranes in 100 µL of reaction buffer, and 125 µL of the tested drug dilution. Non-specific binding was determined in the presence of cocaine, 100 µM for hDAT and paroxetine 3 µM for hSERT, as they are known to be able to fully displace [^3^H]WIN35,428 and [^3^H]imipramine binding, respectively. This non-specific binding also allows subtracting the binding to other components such as the microfiber filters or membrane lipids from the total binding values. Incubation was performed for 1 h at 22 °C. Once the incubation time was over, the binding reactions were stopped by rapid filtration of the membranes through GF/B glass microfiber filters pre-soaked with 0.5 % polyethyleneimine and rapid washing with ice-cold wash buffer (120 nM NaCl, 2 mM MgCl_2_, 10 mM Tris, and 100 μM ZnCl_2_ for hDAT, and 120 nM NaCl, 2 mM MgCl_2_, and 10 mM Tris, for hSERT). The filters were then placed into vials and scintillation cocktail was added. The trapped radioactivity was quantified by liquid scintillation counting. Specific binding of each drug to the transporter was defined as the difference between total binding (binding buffer in absence of drug) and non-specific binding.

### 4.4. Molecular Docking of α-PVP Derivatives at hDAT

The binding mechanism was predicted for the studied compounds by means of molecular docking using MOE 2019.01 software (Molecular Operating Environment, Chemical Computing Group, Montreal, Canada, 2019). The three-dimensional structure of human DAT (hDAT, Uniprot ID: Q01959) protein was predicted by homology modelling using the structure of Drosophila DAT protein as template (PDBID: 4XP6) [63]. This model has already been successfully applied for the study of cathinone’s binding mechanism [14]. Triangle matcher was used as the placement method, and the free energy of binding was predicted using the London dG score function. Th docking protocol was validated by reproducing the interaction mechanism described for cocaine (PDBID:4XP4). Cathinones derivatives were built as (S)-enantiomers in protonated form, and they were docked into the hDAT model using the previous protocol.

### 4.5. Horizontal Locomotor Activity (HLA)

A habituation phase was performed to reduce the novelty and stress associated with handling and injection. In this habituation phase, all mice received, for two consecutive days, an intraperitoneal (i.p.) saline injection and were then placed into a black Plexiglass open field arena (25 × 25 × 40 cm) under low-light conditions and white noise for 30 min. On the test day, the horizontal locomotor activity (HLA) produced by the tested cathinones was measured as described by [14], with minor modifications. Briefly, the animals received the corresponding i.p. injection (saline 5 mL kg^−1^, 3-F-α-PVP, 4-F-α-PVP, 3-Cl-α-PVP, 4-Cl-α-PVP, 3,4-Cl_2_-α-PVP, 3-Br-α-PVP or 4-Br-α-PVP 2.5, 10 or 25 mg kg^−1^ and were immediately placed in the open field arena with the same conditions of light and noise. HLA was video-monitored for 1 h, and their total travelled distance (in cm) was measured using a specific tracking software (Smart 3.0 Panlab, Barcelona, Spain).

### 4.6. Open Field Test: Center vs. Periphery

Open-field experiments are straight-forward tests that evaluate the animals’ activity in response to novelty (or anxiogenic environment) and pharmacological treatments. Each animal was placed into the center of an open field arena, with sufficient height to prevent the animals from escaping (25 cm length × 25 cm width × 40 cm height), and the time spent (in seconds) in the center (8 × 8 cm) or the periphery of the arena was monitored for 60 min (Smart 3.0 Panlab, Barcelona, Spain).

### 4.7. Conditioned Place Preference (CPP)

A place conditioning paradigm was used to determine the potential of the seven α-PVP derivatives to induce rewarding effects. The apparatus that is used for this test consists of two compartments, with differences in tactile and visual cues, communicated by a central corridor. Conditioned place preference (CPP) was performed as described by [61]. Briefly, CPP was performed in three phases: preconditioning test and conditioning and post-conditioning test. During the preconditioning phase, mice had free access to both compartments during 15 min, and the time spent in each compartment was recorded and monitored (Smart 3.0 Panlab, Barcelona, Spain). During the conditioning phase (8 sessions), access to the corridor was closed, and mice received an i.p. injection of the corresponding α-PVP derivative and were immediately placed into one of the compartments for 20 min. The doses tested are in accordance with the ones used in the HLA experiment. On the alternative session, mice were placed for 20 min in the other compartment after receiving a saline i.p. injection. Mice in the control group received a saline injection in every session. The compartment and session in which mice received the drug was randomized. On the test day (post-conditioning phase), the same conditions as in the pre-conditioning phase applied. A preference score, expressed in seconds, was calculated as the difference between the time spent in the drug-paired compartment in the post-conditioning test minus the time spent in the preconditioning phase. An exclusion criterion was predefined for animals that had an initial preference for one of the compartments (>70% of the total session time) in the preconditioning test.

### 4.8. Data and Statistical Analysis

The sample size was determined using GPower software. The minimal significance (α) was set at 0.05 and statistical power at 0.8. Researchers were blinded when performing analysis and data evaluation. Data were expressed as mean ± SEM. Nonlinear regression was used to fit the different competition curves. Data were plotted and best fitted to a sigmoidal dose-response curve, and an IC_50_ value was obtained. Transporter ratios were calculated as (1/DAT IC_50_:1/SERT IC_50_). High ratios indicate greater selectivity for DAT. The Cheng–Prusoff equation was used to calculate *K*_i_ (affinity): *K*_i_ = EC_50_/(1 + [radioligand concentration/*K*_d_]) [64]. One-way or two-way ANOVA of repeated measures, and subsequent Tukey’s post hoc test, conducted only if F was significant, was used to determine the effects of α-PVP derivatives on behavioral experiments. The α-error probability was set at 0.05 (*p* < 0.05). GraphPad Prism software (GraphPad software, San Diego, CA, USA) was used to carry out all the statistic calculations. All the statistical results are included in the Supplemental Material Tables S1–S3 to improve the readability of the manuscript.

## 5. Conclusions

In summary, the present study demonstrates that halogen substitutions in the *meta*- position of α-PVP derivatives, in comparison to their *para*-analogs, slightly increase DA uptake inhibition potency and DAT binding affinity, as well as decrease 5-HT uptake inhibition potency and SERT binding affinity in vitro, triggering an enhanced DAT selectivity. In silico, the loss of interaction with Tyr156 (DAT) of *para*-substituted pyrrolidinophenone derivatives may explain the differential potency and affinity observed in vitro. Moreover, all the compounds tested are more DAT selective than cocaine, pointing to a high abuse liability. The different efficacy inducing hyperlocomotion at the medium dose tested (10 mg kg^−1^) between *meta*- and *para*-analogs appears to be related to an increased DA uptake inhibition and DAT binding, as well as with DAT selectivity. Furthermore, this study demonstrates for the first time the acute anxiogenic and rewarding properties of halogen-ring-derivatives of α-PVP. Therefore, the appearance and use of these halogen-analogs of α-PVP should be monitored, as they seem to be promising new alternatives to α-PVP, especially *meta*-halogen-α-PVP derivatives. Although diverse reports regarding the prevalence of NPS use indicate that most NPS consumers are men [65,66,67], further research is needed in order to identify any sex differences in the psychostimulant and rewarding properties of novel synthetic cathinones. Finally, the present SAR study might provide insights to predict/foretell the effects of novel synthetic cathinones with similar structural changes that may appear in the near future.

## Figures and Tables

**Table 1:** Monoamine uptake inhibition and transporter binding affinities at DAT and SERT of substituted cathinones and cocaine. For monoamine uptake inhibition assays, values are IC_50_, given as μM (mean ± SEM) and for transporter binding affinities assays, values are *Ki*, given as μM (mean ± SEM) of 6 independent experiments performed per triplicate. hDAT/hSERT inhibition and affinity ratios were also calculated, as mentioned in Section 4.

	Transfected HEK293 Cells					
	Monoamine Uptake Inhibition			Transporter Binding Affinities		
Compound	[^3^H]MPP^+^ Uptake at hDAT	[^3^H]5-HT Uptake at hSERT	hDAT/hSERT Inhibition Ratio	[^3^H]WIN35,428 Binding at hDAT	[^3^H]Imipramine Binding at hSERT	hDAT/hSERT Affinity Ratio
**α-PVP**	0.129 (±0.002)	>100	3418	0.017 (±0.001)	>100	8514
**3-F-α-PVP**	0.132 (±0.016)	>100	1819	0.014 (±0.001)	63.53 (±7.62)	4540
**4-F-α-PVP**	0.154 (±0.019)	>100	1366	0.049 (±0.004)	57.88 (±4.27)	1173
**3-Cl-α-PVP**	0.349 (±0.033)	33.33 (±2.46)	95	0.013 (± 0.001)	16.43 (±0.50)	1234
**4-Cl-α-PVP**	0.391 (±0.025)	15.17 (±0.24)	38	0.016 (± 0.001)	7.90 (±0.18)	478
**3,4-Cl-α-PVP**	0.266 (±0.012)	12.74 (±0.28)	47	0.011 (± 0.001)	4.99 (±0.27)	462
**3-Br-α-PVP**	0.117 (±0.016)	39.96 (±6.38)	341	0.012 (±0.002)	5.60 (±0.31)	470
**4-Br-α-PVP**	0.156 (±0.012)	23.11 (±2.27)	148	0.024 (±0.001)	1.25 (±0.09)	52
**Cocaine**	0.231 (±0.013)	1.85 (±0.28)	7.98	0.283 (±0.04)	0.54 (±0.03)	1.92

hDAT/hSERT ratio = 1/DAT IC_50_:1/SERT IC_50_.

## Data Availability

The data that support the findings of this study are available from the corresponding author upon reasonable request.

## Supplementary Materials

The following supporting information can be downloaded at: https://www.mdpi.com/article/10.3390/ijms23042226/s1.
