# Supplementary material for: Neuropsychopharmacology of Emerging Drugs of Abuse: meta- and para-Halogen-Ring-Substituted α-PVP (“flakka”) Derivatives
Source: Int J Mol Sci. 2022 Feb 17;23(4):2226. doi: 10.3390/ijms23042226 (PMC8876800; doi:10.3390/ijms23042226)
Supplement: Supplementary file 1 [file ijms-23-02226-s001.zip › ijms-1580528-supplementary.pdf]

Supplemental Material

Neuropsychopharmacology of emerging drugs of abuse: *meta*- and *para*-halogen-ring-substituted α-PVP ("*flakka*") derivatives.

Núria Nadal-Gratacós, Esther Lleixà, Monica Gibert-Serramià, Roger Estrada-Tejedor, Xavier Berzosa, Xavier Batllori, David Pubill, Jordi Camarasa, Elena Escubedo , Raúl López-Arnau.

Synthesis of α-PVP derivatives

The synthesis and characterization of the synthetic cathinones was carried out through three steps, following the procedure formerly described by (Meltzer et al., 2006), with minor modifications. The ketone intermediate (**2**) was synthesized as a result of the reaction between the corresponding ring-substituted nitrile (**1**) compound with n-Butylmagnesium chloride (n-BuMgCl), in anhydrous conditions, followed by acidic hydrolysis (Step 1). Generally, after 6 h at room temperature, the reaction was completed. The reaction mixture was cooled, and a 5% solution of sulfuric acid (H_2SO_4) was added drop by drop into the reaction mixture. Phases were separated, the aqueous layer was extracted with diethyl ether (Et_2O), the organic phases were combined, dried with magnesium sulfate ($MgSO_4$), filtered and reduced in vacuo to an oil. To achieve the α-bromination of the ketone intermediate (Step 2), bromine (Br_2) was added in a dropwise manner to a solution of **2** in dichloromethane (CH_2Cl_2) in presence of acetic acid (AcOH) in catalytic amounts. After 1.5-6 h the reaction was completed. The excess of bromine was neutralized with a 10% aqueous solution of sodium thiosulfate ($Na_2S_2O_3$). The organic layer was separated, washed with a 0,01M solution of NaOH, dried ($MgSO_4$), filtrated and reduced in vacuo to an oil. Column chromatography was performed in order to isolate the α-bromoketone (**3**). Pyrrolidine was dissolved in ethanol (EtOH), and **3** was added in a drop-wise manner (Step 3). It was allowed to react at room temperature. After 1-4 h the reaction was completed. The reaction mixture was washed with water, extracted with hydrochloric acid (HCl) 1 N in water and then back-extracted into Et_2O by basification to pH 10 with sodium hydroxide (NaOH) 1 N. The organic layer was dried with $MgSO_4$, filtered and reduced in vacuo to an oil. The resulting oil was dissolved in EtOH and a mixture of Et_2O with HCl (3 M) in cyclopentyl methyl ether (CPME) was added in a dropwise manner to obtain the hydrochloride salt (**4**). Solids were collected by filtration. The identification of the seven synthesized cathinones was assessed by thin layer chromatography (TLC), proton and carbon nuclear magnetic resonance (^{1}H NMR, ^{13}C NMR), infrared spectroscopy (IR) and liquid chromatography-mass spectrometry (LC/MS) yielding the following results:

3-F-α-PVP:

^{1}H-NMR (400 MHz, Chloroform-*d*) δ: 12.80 (s, 1H), 7.74 (d, *J* = 7.8 Hz, 1H), 7.65 (dt, *J* = 9.1, 1.7 Hz, 1H), 7.56 (td, *J* = 8.0, 5.4 Hz, 1H), 7.41 (td, *J* = 8.1, 2.3 Hz, 1H), 5.07 (m, *J* = 8.9, 4.8 Hz, 1H), 3.85 – 3.77 (m, 2H), 3.70 – 3.64 (m, 1H), 2.84 – 2.77 (m, 1H), 2.29 – 2.12 (m, 4H), 2.06 – 1.98 (m, 2H), 1.50 (m, *J* = 13.3, 6.9 Hz, 1H), 1.33 (m, *J* = 20.1, 6.9 Hz, 1H), 0.93 (t, *J* = 7.3 Hz, 3H).

^{13}C NMR (100 MHz, Chloroform-*d*) δ: 195.94 , 195.91 , 164.50 , 162.00 , 137.93 , 137.86 , 131.45 , 131.37 , 124.53 , 124.50 , 122.63 , 122.415 , 115.46 , 115.23 , 62.52 , 53.08 , 49.15 , 33.07 , 24.16 , 23.90 , 19.90 , 14.12 .

IR (KBr), v max: 3417 , 2962 , 1689 , 1448 , 1258 , 1019 , 754 cm^{-1}.

m/z: 250 (M+)

4-F-α-PVP:

^{1}H-NMR (400 MHz, Chloroform-*d*) δ: 12.64 (s, 1H), 8.03 (dd, *J* = 8.6, 5.2 Hz, 2H), 7.23 (t, *J* = 8.4 Hz, 2H), 5.16 – 5.09 (m, 1H), 3.86 – 3.77 (m, 2H), 3.66 (dd, *J* = 9.9, 5.9 Hz, 1H), 2.84 – 2.77 (m, 1H), 2.27 – 2.11 (m, 4H), 2.05 – 1.96 (m, 2H), 1.48 (dt, *J* = 13.2, 7.2 Hz, 1H), 1.32 (dt, *J* = 13.2, 6.8 Hz, 1H), 0.92 (t, *J* = 7.3 Hz, 3H).

^{13}C NMR (100 MHz, Chloroform-d) δ 195.36 , 168.36 , 132.40 , 131.70 , 131.60 , 117.04 , 116.82 , 62.30 , 53.02 , 49.19 , 33.09 , 24.15 , 23.88 , 19.86 , 14.12 .

IR (KBr), v max: 3437 , 2964 , 1682 , 1440 , 1234 , 1006 , 743 cm^{-1}.

m/z: 250 (M+)

3-Cl-α-PVP:

^{1}H-NMR (400 MHz, Chloroform-*d*) δ: 12.64 (s, 1H), 7.93 (t, *J* = 1.8 Hz, 1H), 7.87 (dt, *J* = 7.8, 1.2 Hz, 1H), 7.66 (ddd, *J* = 8.0, 2.1, 0.8 Hz, 1H), 7.51 (t, *J* = 7.9 Hz, 1H), 5.15 (dt, *J* = 9.0, 5.3 Hz, 1H), 3.85 – 3.74 (m, 2H), 3.68 (dt, *J* = 10.0, 5.7 Hz, 1H), 2.84 (dd, *J* = 10.3, 7.3 Hz, 1H), 2.28 – 2.11 (m, 4H), 2.06 – 1.97 (m, 2H), 1.52 – 1.43 (m, 1H), 1.32 (dd, *J* = 13.4, 6.5 Hz, 1H), 0.91 (t, *J* = 7.3 Hz, 3H).
^{13}C NMR (100 MHz, Chloroform-d) *δ:* 195.93 , 137.32 , 136.11 , 135.20 , 130.90 , 1328.59 , 126.86 , 62.84 , 53.06 , 49.46 , 33.01 , 24.14 , 23.89 , 19.90 , 14.12
IR (KBr), v max: 3424 , 2961 , 1689 , 1451 , 1228 , 754 cm^{-1}.
m/z: 266 , 268 (M+, M+2)
4-Cl-α-PVP:
^{1}H-NMR (400 MHz, Chloroform-*d)* δ: 12.55 (s, 1H), 7.96 – 7.92 (m, 2H), 7.55 – 7.49 (m, 2H), 5.20 (dt, *J* = 9.0, 5.1 Hz, 1H), 3.79 (ddd, *J* = 14.8, 10.8, 6.2 Hz, 2H), 3.67 (dd, *J* = 10.1, 6.1 Hz, 1H), 2.85 (dd, *J* = 10.3, 7.4 Hz, 1H), 2.26 – 2.11 (m, 4H), 2.05 – 1.96 (m, 2H), 1.46 (dt, *J* = 13.2, 6.6 Hz, 1H), 1.32 (dt, *J* = 13.4, 6.8 Hz, 1H), 0.90 (t, *J* = 7.3 Hz, 3H).
^{13}C NMR (100 MHz, Chloroform-d) δ: 195.85 , 142.17 , 134.16 , 130.13 , 129.95 , 62.64 , 53.00 , 49.44 , 33.03 , 24.13 , 23.87 , 19.81 , 14.12
IR (KBr), v max: 3430 , 2962 , 1684 , 1439 , 1232 , 754 cm^{-1}.
m/z: 266 , 268 (M+, M+2)
3,*4-Cl$_2$-α-PVP*:
^{1}H-NMR (400 MHz, DMSO-*d6)* δ: 10.75 – 10.66 (m, 1H), 8.34 (d, *J* = 2.0 Hz, 1H), 8.03 (dd, *J* = 8.4, 2.1 Hz, 1H), 7.93 (d, *J* = 8.4 Hz, 1H), 5.57 (dt, *J* = 8.5, 5.5 Hz, 1H), 3.64 – 3.59 (m, 1H), 3.47 (t, *J* = 6.6 Hz, 1H), 3.23 – 3.16 (m, 1H), 3.10 (dd, *J* = 11.5, 6.7 Hz, 1H), 2.08 – 1.96 (m, 2H), 1.96 – 1.89 (m, 4H), 1.26 (dd, *J* = 14.9, 7.6 Hz, 1H), 1.06 – 0.98 (m, 1H), 0.78 (t, *J* = 7.2 Hz, 3H).
^{13}C NMR (100 MHz, DMSO-*d6*) δ: 195.01 , 137.85 , 134.55 , 132.34 , 131.64 , 130.76 , 128.79 , 67.50 , 53.71 , 51.96 , 31.40 , 22.94 , 17.25 , 13.68 .
IR (KBr), v max: 3430 , 2931 , 1689 , 1449 , 1217 , 748.
m/z: 300 , 302(M+, M+2)
3-Br-α-PVP:
^{1}H-NMR (400 MHz, Chloroform-*d)* δ: 12.81 (s, 1H), 8.08 (t, *J* = 1.8 Hz, 1H), 7.88 (ddd, *J* = 7.9, 1.8, 1.0 Hz, 1H), 7.82 (ddd, *J* = 8.0, 2.0, 0.9 Hz, 1H), 7.45 (t, *J* = 7.9 Hz, 1H), 5.04 (t, *J* = 6.7 Hz, 1H), 3.80 (m, 2H), 3.67 (m, 1H), 2.78 (m, 1H), 2.32 – 2.12 (m, 4H), 2.06 – 1.94 (m, 2H), 1.55 – 1.42 (m, 1H), 1.39 – 1.25 (m, 1H), 0.93 (t, *J* = 7.3 Hz, 3H).
^{13}C NMR (100 MHz, Chloroform-d) δ: 195.87 , 138.19 , 137.58 , 131.55 , 131.11 , 127.14 , 124.08 , 62.23 , 53.08 , 49.00 , 33.06 , 24.16 , 23.90 , 19.94 , 14.13 .
IR (KBr), v max: 3410 , 2960 , 1689 , 1451 , 1225 , 750.
m/z: 310, 312 (M+, M+2)
4-Br-α-PVP:
^{1}H-NMR (400 MHz, Chloroform-*d)* δ: 12.71 (s, 1H), 7.83 (d, *J* = 8.7 Hz, 2H), 7.71 (d, *J* = 8.6 Hz, 2H), 5.09 – 5.04 (m, 1H), 3.84 – 3.77 (m, 2H), 3.66 (dd, *J* = 10.5, 5.1 Hz, 1H), 2.78 (dd, *J* = 10.2, 7.4 Hz, 1H), 2.27 – 2.12 (m, 4H), 2.04 – 1.97 (m, 2H), 1.51 – 1.44 (m, 1H), 1.32 (dd, *J* = 12.5, 6.3 Hz, 1H), 0.92 (t, *J* = 7.3 Hz, 3H).
^{13}C NMR (100 MHz, Chloroform-d) δ: 196.10 , 134.63 , 134.58 , 133.00 , 130.05 , 62.11 , 53.02 , 49.05 , 33.06 , 24.16 , 23.88 , 19.93 , 14.12 .
IR (KBr), v max: 3429 , 2961 , 1684 , 1440 , 1231 , 749.
m/z: 310, 312 (M+, M+2)

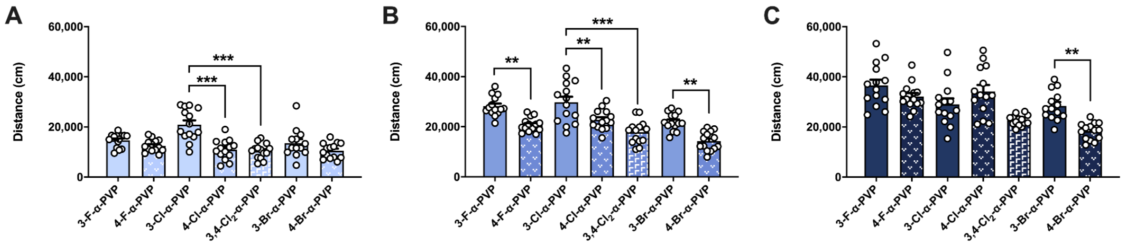

Figure S1. Effects of halogenated α-PVP derivatives at 2.5 mg kg^{-1} (A), 10 mg kg^{-1} (B) and 25 mg kg^{-1} (C) on cumulative locomotor activity in CD-1 mice. Bars represent mean ± SEM of the distance travelled in 60 min. Panel A: 3-F-α-PVP, 4-F-α-PVP, 3-Cl-α-PVP, 4-Cl-α-PVP, 3,4-Cl_2-α-PVP and 4-Br-α-PVP 2.5 mg kg^{-1} N=14/group and 3-Br-α-PVP N=13/group. Panel B and C: N=14/group. One-way ANOVA yielded a significant effect of the variable *Drug* for all the doses tested (2.5 mg kg^{-1} $F_{(6,90)}=11.38$; $p<0.001$, 10 mg kg^{-1} $F_{(6,91)}=18.71$; $p<0.001$ and 25 mg kg^{-1} $F_{(6,91)}=12.95$; $p<0.001$). **$p<0.01$ and ***$p<0.001$ vs meta-analog.

Table S1. Statistical results of HLA (One-way ANOVA) and HLA time course (Two-way ANOVA of repeated measures).

		Statistical results (F and p value)	
**Compound**	**Variables**	**HLA**	**HLA time course**
**3-F-α-PVP**	*Dose*	$F_{(3, 52)} = 91.16$; $p<0.001$	$F_{(3, 52)} = 91.16$; $p<0.001$
	Time	N.A.	$F_{(11, 572)} = 14.89$; $p<0.001$
	Interaction	N.A.	$F_{(33, 572)} = 6.459$; $p<0.001$
**4-F-α-PVP**	*Dose*	$F_{(3, 52)} = 131.6$; $p<0.001$	$F_{(3, 52)} = 146.9$; $p<0.001$
	Time	N.A.	$F_{(11, 572)} = 75.43$; $p<0.001$
	Interaction	N.A.	$F_{(33, 572)} = 7.317$; $p<0.001$
**3-Cl-α-PVP**	*Dose*	$F_{(3, 52)} = 31.17$; $p<0.001$	$F_{(3, 52)} = 30.83$; $p<0.001$
	Time	N.A.	$F_{(11, 572)} = 34.53$; $p<0.001$
	Interaction	N.A.	$F_{(33, 572)} = 3.613$; $p<0.001$
**4-Cl-α-PVP**	*Dose*	$F_{(3, 51)} = 52.82$; $p<0.001$	$F_{(3, 51)} = 52.82$; $p<0.001$
	Time	N.A.	$F_{(11, 561)} = 9.291$; $p<0.001$
	Interaction	N.A.	$F_{(33, 561)} = 2.078$; $p<0.001$
**3,4-Cl_2-α-PVP**	*Dose*	$F_{(3, 52)} = 57.09$; $p<0.001$	$F_{(3, 52)} = 57.07$; $p<0.001$
	Time	N.A.	$F_{(11, 572)} = 73.67$; $p<0.001$
	Interaction	N.A.	$F_{(33, 572)} = 10.13$; $p<0.001$
**3-Br-α-PVP**	*Dose*	$F_{(3, 51)} = 45.83$; $p<0.001$	$F_{(3, 51)} = 45.83$; $p<0.001$
	Time	N.A.	$F_{(11, 561)} = 57.06$; $p<0.001$
	Interaction	N.A.	$F_{(33, 561)} = 5.505$; $p<0.001$
**4-Br-α-PVP**	*Dose*	$F_{(3, 52)} = 23.00$; $p<0.001$	$F_{(3, 52)} = 23.00$; $p<0.001$
	Time	N.A.	$F_{(11, 572)} = 93.55$; $p<0.001$
	Interaction	N.A.	$F_{(33, 572)} = 5.529$; $p<0.001$

N.A., not assessed

Table S2. Statistical results (One-way ANOVA) of Open Field Test.

Compound	Variable	Statistical results (F and p value) Open Field Test
**3-F-α-PVP**	*Dose*	$F_{(3, 36)} = 12.33$; $p<0.001$
**4-F-α-PVP**	*Dose*	$F_{(3, 36)} = 4.864$; $p<0.01$
**3-Cl-α-PVP**	*Dose*	$F_{(3, 36)} = 12.24$ $p<0.001$
**4-Cl-α-PVP**	*Dose*	$F_{(3, 36} = 6.606$; $p<0.01$
**3,4-Cl_2-α-PVP**	*Dose*	$F_{(3, 36)} = 8.244$; $p<0.001$
**3-Br-α-PVP**	*Dose*	$F_{(3, 36)} = 7.007$; $p<0.001$
**4-Br-α-PVP**	*Dose*	$F_{(3, 36)} = 1.726$; $p>0.05$

Table S3. Statistical results (One-way ANOVA) of CPP**.**

Compound	Variable	Statistical results (F and p value)
		**CPP**
**3-F-α-PVP**	*Dose*	$F_{(3, 51)} = 7.272$; $p<0.001$
**4-F-α-PVP**	*Dose*	$F_{(3, 50)} = 10.46$; $p<0.001$
**3-Cl-α-PVP**	*Dose*	$F_{(3, 52)} = 7,175$; $p<0.001$
**4-Cl-α-PVP**	*Dose*	$F_{(3, 49)} = 6.090$; $p<0.01$
**3,4-Cl$_2$-α-PVP**	*Dose*	$F_{(3, 51)} = 7,858$; $p<0.001$
**3-Br-α-PVP**	*Dose*	$F_{(3, 48)} = 6.656$; $p<0.001$
**4-Br-α-PVP**	*Dose*	$F_{(3, 49)} = 14.93$; $p<0.001$
